# Imaging of Pathologies of the Temporal Bone and Middle Ear: Inflammatory Diseases, Their Mimics and Potential Complications—Pictorial Review

**DOI:** 10.3390/tomography9060170

**Published:** 2023-12-08

**Authors:** Christopher Kloth, Annika Beck, Nico Sollmann, Meinrad Beer, Marius Horger, Wolfgang Maximilian Thaiss

**Affiliations:** 1 Department of Diagnostic and Interventional Radiology, University Hospital Ulm, Albert-Einstein-Allee 23, 89081 Ulm, Germany; nico.sollmann@uniklinik-ulm.de (N.S.); meinrad.beer@uniklinik-ulm.de (M.B.); wolfgang.thaiss@uniklinik-ulm.de (W.M.T.); 2 Radiology and Radiation Therapy Lindau, Friedrichshafener Str. 83, 88131 Lindau (Lake Constance), Germany; 3 Institute of Pathology, University Hospital Ulm, Albert-Einstein-Allee 23, 89081 Ulm, Germany; annika.beck@uniklinik-ulm.de; 4 Department of Diagnostic and Interventional Neuroradiology, School of Medicine, Klinikum Rechts der Isar, Technical University of Munich, Ismaninger Str. 22, 81675 Munich, Germany; 5 TUM-Neuroimaging Center, Klinikum Rechts der Isar, Technical University of Munich, 81675 Munich, Germany; 6 Department of Diagnostic and Interventional Radiology, Eberhard-Karls-University, Hoppe-Seyler-Str. 3, 72076 Tuebingen, Germany; marius.horger@med.uni-tuebingen.de; 7 Department of Nuclear Medicine, University Hospital Ulm, Albert-Einstein-Allee 23, 89081 Ulm, Germany

**Keywords:** temporal bone, ear, external auditory canal, middle ear, inner ear, inflammation, otitis, otitis externa maligna, cholesteatoma, imaging, computed tomography, magnetic resonance imaging

## Abstract

Imaging of the temporal bone and middle ear is challenging for radiologists due to the abundance of distinct anatomical structures and the plethora of possible pathologies. The basis for a precise diagnosis is knowledge of the underlying anatomy as well as the clinical presentation and the individual patient’s otological status. In this article, we aimed to summarize the most common inflammatory lesions of the temporal bone and middle ear, describe their specific imaging characteristics, and highlight their differential diagnoses. First, we introduce anatomical and imaging fundamentals. Additionally, a point-to-point comparison of the radiological and histological features of the wide spectrum of inflammatory diseases of the temporal bone and middle ear in context with a review of the current literature and current trends is given.

## 1. Introduction, Anatomy and Clinical Features

The petrous bone represents a part of the temporal bone, which consists of the pars squamosa, pars tympanica, and pars mastoidea [1]. It includes the inner ear, the internal auditory canal, and the petrous bone itself [2,3]. Anatomically, the middle ear and mastoid already belong to the pars tympanica and pars mastoidea, which could be depicted by imaging [1]. In this context, imaging should ideally be evaluated in conjunction with a detailed patient history, especially including information on previous surgery, presence of chronic infections, possible anti-inflammatory medication, and the current otoscopic findings. The imaging consideration and description should always be carried out systematically, e.g., from the outside inwards.

The external ear includes the auricle and external auditory canal (EAC), which extends to the tympanic membrane [1]. Anatomically the lateral third of the EAC is fibrocartilaginous, while the medial two-thirds of the EAC are surrounded by the temporal bone [1].

The middle ear is located next to the external ear canal. The middle ear is an air-filled cavity within the petrous portion of the temporal bone that contains the ossicular chain [1]. Key anatomical structures of the middle ear comprise (a) the upper air-filled part of the middle ear (epitympanum, dome space); (b) the middle space (mesotympanum) at the level of the eardrum; and (c) the hypotympanum below the level of the external acoustic canal [4]. Within the middle ear, other relevant landmarks are the scutum, Prussak’s space, tegmen tympani as the roof of the middle ear, and the tegmen mastoideum as the roof of the mastoid. Particularly in the case of the frequent pathology of cholesteatoma, the preferred localization in the epitympanum must always be examined closely.

The vestibulocochlear nerve and the facial nerve are the most important nerval structures. In the case of the facial nerve, the individual stations and courses in the petrous bone are of immanent importance. Beginning in the cerebellopontine angle (a), the nerve runs through the internal auditory canal (b) to the labyrinthine section (c) where the first nerve branches are released at the first genu. The nerve then runs through the tympanum (d) along the tympanic cavity via the mastoid (e) to the stylomastoid foramen (f) [1,2,3]. Furthermore, the inner ear includes the cochlea, vestibule, and the semicircular canals.

Across these anatomical structures, different inflammatory processes can occur [5]. Infection is a key cause of inflammation in the temporal bone; however, important mimics must be considered [5]. Those will be discussed in this article. Furthermore, we aimed to summarize the most common inflammatory lesions of the temporal bone and middle ear, to describe their specific imaging characteristics, and their differential diagnoses in order to distinguish them from chronification and complications.

## 2. Imaging

There is a wide range of imaging methods for assessing the temporal bone. Due to the widespread use of cross-sectional imaging methods, computed tomography (CT) is the most used modality, not only for inflammatory changes, but also for tumors or preoperative imaging and visualization of anatomical structures [6,7,8]. Targeted X-rays in Schuller’s, Stevener’s, or Mayer’s view are only used in rare cases. In addition to the acute scenario of the assessment of a temporal bone trauma, CT is used for the assessment of otological status by acquisitions with very thin slices. The complete petrous roof should be imaged cranially, and the complete mastoid process covered caudally. The slice thickness should not exceed 0.5–1 mm.

Indications for magnetic resonance imaging (MRI) can be the localization of a fascial nerve damage or bleeding into the labyrinthine system [9]. Here, attention must always be paid to acquiring thin slices with a sufficiently high matrix. Bone involvement can be detected with MRI through bone marrow edema; however, early bone erosions and destruction are better visualized with CT imaging. Additionally, MRI is helpful for any form of tumor imaging and for further visualization of anatomical structures [10].

## 3. Clinical Features and Differential Diagnoses

An overview of the most common differential diagnoses for inflammatory diseases of the temporal bone and middle ear are given in Table 1. The most important entities are presented systematically from the periphery to the center of the inner ear in detail.

### 3.1. External Auditory Canal

#### 3.1.1. Otitis Externa Maligna

Otitis externa maligna can also be called otitis externa necroticans. The term “maligna” is due to the aggressive clinical course. This is an infectious and mostly necrotizing inflammation of the auditory canal. There can be adjacent destruction of bone and soft tissue. The clinical features are a swollen EAC and significant ear pain [22], and otalgia is commonly the leading symptom. Most commonly, only one side is affected; however different cases with bilateral manifestations have been reported [23].

Predisposing factors are immunosuppression and diabetes, and the prevalence is higher among elderly patients. Pseudomonas aeruginosa is of particular importance as a causative pathogen, which should always be confirmed by otoscopic swabs [1,22]. Pathogenesis is unknown, and ischemic conditions due to diabetic microangiopathy have been discussed [22,23,24]. There is usually a chain of inflammatory progression from initial cellulitis to chondritis, followed potentially by osteitis and finally osteomyelitis [1]. A granulating inflammation with exposed bone parts can be seen as the otoscopic finding. In case of bony skull involvement, a progressive replacement of compact bone with granulation tissue is registered [1].

**Figure 1:**
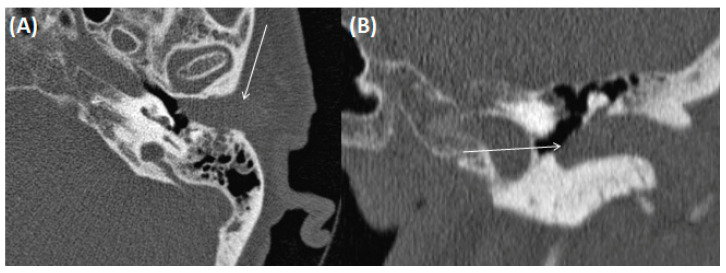
CT of a 73-year-old female patient in axial (**A**); and coronal orientation (**B**). Due to complete soft tissue obstruction of the external auditory canal, otitis externa maligna was assumed. However, after biopsy a squamous cell carcinoma was confirmed.

An abscess, osteomyelitis, or a tumor must always be considered as differential diagnoses (Figure 1). In this case, MRI can be particularly helpful; a low signal on T1-weighted imaging makes an osteomyelitic component more likely [22,24]. Furthermore, CT can show a subtotal obstruction of the meatus acusticus externus with bony wall erosions [22,23,24]. Imaging is necessary to determine the extent of the destruction. Involvement and infiltration of the adjacent anatomical structures is possible: caudal involvement of the parotid gland and masticator cavity, posterior into the mastoid cells, medial into the middle ear, or cranial to the temporomandibular joint [22,23,24]. Therapy includes the spectrum of radical removal of the inflammatory tissue, antibiotic therapy, and aural toilet [23]. If an abscess is present, it needs to be resected in total. Here, imaging plays a key role that can help to identify the surrounding soft tissue and adjacent bone structures in order to plan the distinct extent of any possible surgery.

**Figure 2:**
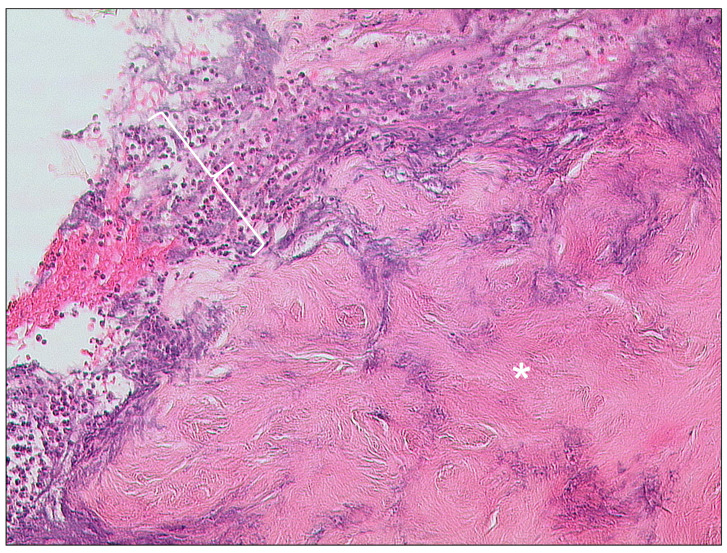
Otitis externa maligna, HE, original magnification 200:1. Necrotic soft tissue (asterisk) with an infiltrate of neutrophilic granulocytes (bracket) in the upper left.

Otitis externa maligna is characterized by necrosis of soft tissue and bone with ulceration of the covering epithelium (Figure 2) [25]. Indicators for a poor prognostic outcome are intracranial extension and involvement of multiple cranial nerves [1].

#### 3.1.2. Cholesteatoma of the External Ear Canal

The EAC cholesteatoma (EACC) has an incidence of approximately 0.1% to 0.5% [26]. Most cases are spontaneous or can arise after previous trauma, surgery, or radiation. Clinically, dull pain and unilateral otorrhea are present. Most of the patients are between 40 and 70 years of age. It is defined as a cystic mass of skin debris and cholesterol crystals that obstructs the EAC [27].

**Figure 3:**
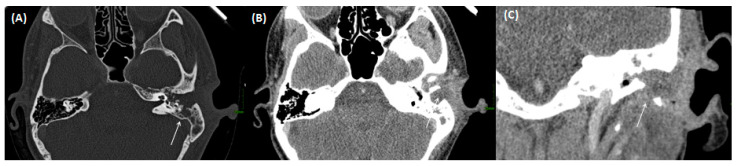
CT of a 26-year-old female patient with axial images using bone (**A**) and soft tissue (**B**) windowing, and coronal orientation using soft tissue windowing (**C**). A cholesteatoma is demarked as a soft tissue focus within the EAC with associated erosions at the border to the mastoidal cells.

**Figure 4:**
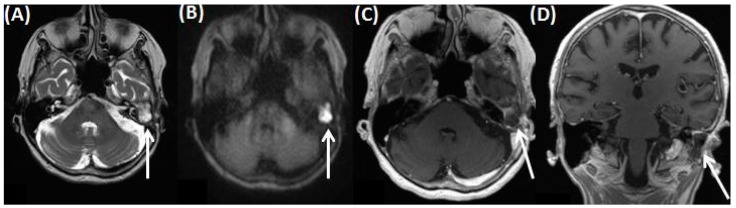
MRI of an 80-year-old female patient with diagnosis of a cholesteatoma of the left EAC after mastoidectomy at this side. Cholesteatoma can be detected as hyperintense on T2-weighted images (**A**), it appears hyperintense on DWI (**B**), and can only be clearly delineated on T1-weighted images after contrast agent application as shown in (**C**,**D**).

Histopathologically, keratinized squamous epithelium can be found along the EAC and signs of local invasion with bony destruction, wandering erosions, and periostitis can be present [28,29]. The tympanic membrane is typically normal. In CT, the cholesteatoma shows a soft tissue focus within the EAC (typically at the inferior wall), with associated erosions (Figure 3) [26]. However, the imaging findings are non-specific and can appear similar in other tumorous diseases (e.g., squamous cell carcinoma). Diffusion-weighted imaging (DWI) from MRI is of particular importance, both in the primary diagnosis and in the diagnosis of recurrence (Figure 4). The benefit of imaging is an additional gain in anatomical information for the surgeon for optimal preparation.

In general, different DWI techniques are used, which basically can be divided into two subtypes: echo-planar imaging (EPI)-based and non-EPI-based techniques [30]. Single-shot EPI-DWI can be seen as a widely available standard DWI technique, which is robust and relatively insensitive to motion artifacts [30]. Cholesteatoma appears hyperintense on DWI, whereas granulation tissue or fibrous tissue has a low signal intensity on DWI (at a b-factor of 800 s/mm^2^) [30,31,32,33,34]. The reason is not fully understood but may be explained by a combination of T2 shine-through and diffusion restriction effects in cholesteatoma [5,31,35]. Non-EPI-based sequences reduce most susceptibility artifacts and permit thinner sections, resulting in improved sensitivity of 90–100% for lesions as small as 2 mm [31]. In this context, the most common cause for missing residual cholesteatoma on DWI sequences is a small lesion size of <4 mm [31].

The principles of treatment for EACC are complete resection, ideally maintaining the normal structure and function of the EAC [27].

#### 3.1.3. Auditory Canal Exostosis

**Figure 5:**
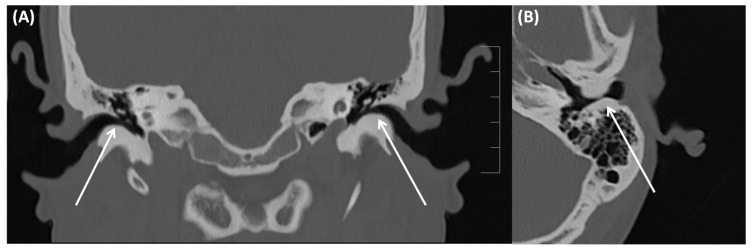
CT of a 48-year-old male patient with a long history of surfing and sailing. The patient was mostly asymptomatic, reporting problems with water getting into the ear that drains poorly. Coronal image (**A**), and axial image (**B**) using bone windowing.Exostosis of the EAC on both sides can be identified (arrows).

This inflammatory condition is also called external otitis. This form of disease can lead to an increased formation of bone substance in the bony part of the EAC (Figure 5). Exostosis of the EAC is a benign bony overgrowth of the bony borders of the auditory canal [36,37,38]. In terms of appearance, the additive bone substance is typically arranged symmetrically on both sides and in a broad-based, circumferential manner. The patients present with a progressive bilateral hearing loss [38]. Due to the possible development after long-term exposure to cold water, the disease is also referred to as “Surfer’s Ear”. An association with cold water or wind is present in up to 80% of the patients [39,40]. There is a strong male predilection registered, which may be related to the preponderance of male participation in the associated sport activities [39,40]. Histopathologically, localized hyperplasia of compact bone is registered, which is the consequence of prolonged vasodilatation following cold water exposure [41]. In most cases, imaging studies are not required because diagnoses can be carried out by clinical examination and inspection [42].

The decision to perform imaging is made on a case-by-case basis, possibly also to rule out other accompanying pathologies or to clearly visualize the anatomical (neighborhood) conditions. However, dedicated imaging is often not necessary. Nevertheless, knowledge of the entity is important, also in order to detect it as a possible coincidence. Cholesteatoma can be ruled out with imaging, which is usually possible due to the unilateral occurrence. In the acute form of external otitis, surrounding cellulitis can also be present, thereby requiring antibiotic treatment.

#### 3.1.4. Keratosis Obturans

**Figure 6:**
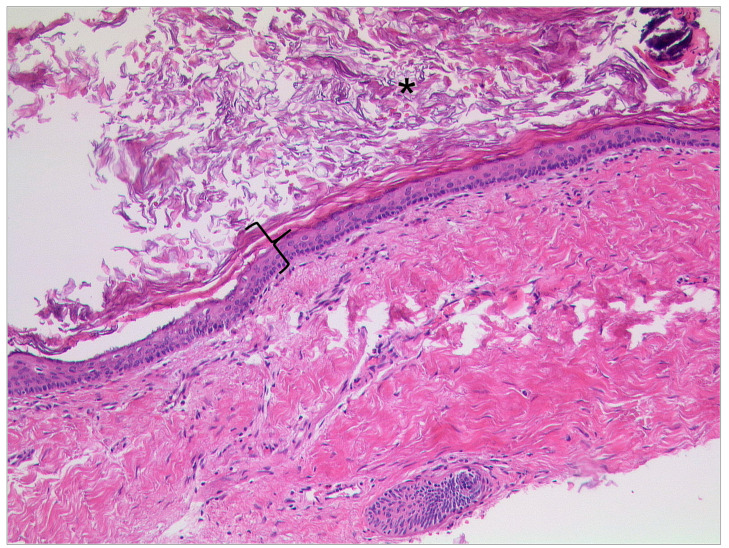
Keratosis obturans: Cutis with massive hyperkeratosis (bracket) and accumulation of keratin (asterisk) on the surface. In this case, the epithelium is rather atrophic instead of the typical acanthosis. Also, the usually present inflammatory infiltrate is lacking in this case. HE, original magnification 100:1.

Keratosis obturans is defined as an expansile accumulation of keratin debris within the EAC [1]. It is characterized by subsequent occlusion and expansion of the bony portion of the EAC by a plug of desquamated keratin. Keratosis obturans is characterized by hyperplasia of the underlying epithelium and a chronic inflammation within the subepithelial tissue (Figure 6) [43,44,45]. 

It can typically be observed in younger patients and it occurs bilaterally in contrast to cholesteatomas. Symptoms of the patients are unspecific pain and conductive hearing loss. On CT images, a diffuse widening of the EAC by an epidermal plug can be registered [1]. The most important differential diagnosis is EAC cholesteatoma; however, bone erosions are typically missing. Total resection is the treatment of choice; however, a high incidence of recurrence is registered. Complications are rare, but facial nerve palsy and dehiscence of adjacent bone structures have been reported in recent literature [46].

### 3.2. Middle Ear and Mastoid

#### 3.2.1. Acute/Chronic Otitis Media

The term otitis media is not very well defined and can be regarded as an umbrella term for various chronic inflammatory subtypes [14]. In the classic sense, it is mostly used for catarrhal inflammation of the Eustachian tube and inner ear in children. The infection is usually caused by bacteria such as Streptococcus species, Haemophilus influenza, or even Moraxella catarrhalis [26].

The term can also include otitis media mesotympanalis, with the peak occurrence of the disease in childhood. In addition to a persistent lack of ventilation of the tympanic space, there is an abundance of mucus. Otitis media in particular is a primary disease of infants and young children. They present with fever, earache, and typically a red, tense eardrum [26]. Imaging is usually not required for uncomplicated acute otitis media [1]. If imaging is performed, opacities in the middle ear and mastoid can be registered, and detection of fluid levels is possible [1].

**Figure 7:**
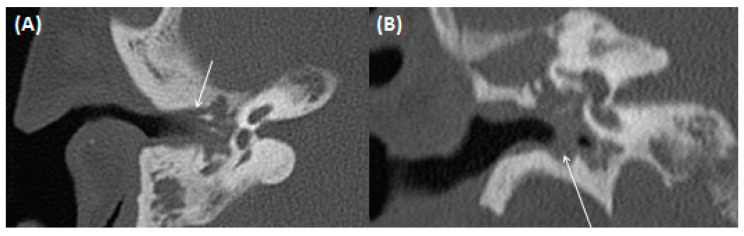
CT of an 84-year-old female patient with axial (**A**) and coronal (**B**) bone window images of the right middle ear. Complete obstruction of the middle ear including the epi-, meso-, and hypotympanum is shown. Images also show a swollen EAC, and the eardrum cannot be clearly defined. Only faint delineation of the auditory ossicles is observed. Bony thinning from the cortex to the base of the skull without clear interruption is present as well.

In otoscopy, the eardrum appears perforated with evidence of secretion [47,48]. In this regard, CT can show a subtotally dislocated tympanic membrane and debris without contrast enhancement (Figure 7). The tympanic membrane may be protruded when secretion is dominant or retracted depending on the presence of catarrh, while the scutum appears intact. Furthermore, MRI typically shows a high signal on T2-weighted imaging and no contrast enhancement, and it is useful to rule out intracranial complications [49,50]. Imaging could help to secure the diagnosis, estimate the extent, and identify potential complications.

Histopathologically, acute mastoiditis is characterized by an infiltrate of neutrophilic granulocytes. Mucosal inflammations of the Eustachian tube, the middle ear, and the tympanic membrane can be registered. Antibiotic therapy is sufficient in most of the patients, but in case of complications a surgical approach can be necessary.

Clinical evidence of complicated mastoiditis may be the presence of postauricular erythema (indicative of Bezold’s abscess) or surrounding edema. In this clinical situation, imaging is recommended to account for possible complications. Other potential complications are subperiostal abscess, coalescent mastoiditis, or an otogenic intracranial abscess with or without associated meningitis. In particular, increasing headache near the affected ear can be a warning sign for intracranial complications.

#### 3.2.2. (Oto-)Mastoiditis

This disease presents as an acute or chronic inflammation of the middle ear with associated bony destruction of the adjacent mastoid [51]. The persistent inflammatory process leads to hyperemia with simultaneous venous stasis. Osteoclast activity is stimulated and leads to bone resorption. Clinically, pronounced earache and signs of inflammation are dominant. The therapy consists of antibiotics and, if not sufficient, subsequent excavation of the mastoid.

**Figure 8:**
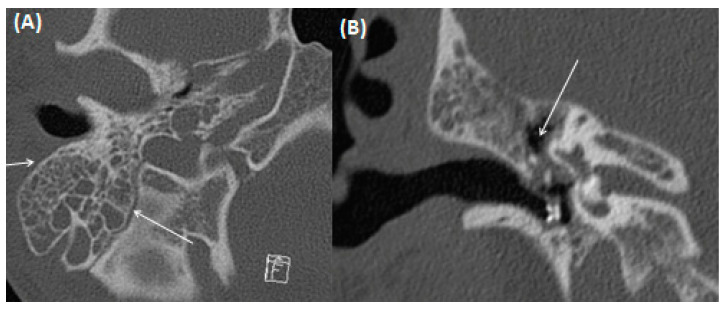
CT of a 17-year-old male patient using bone windowing with axial (**A**) and coronal (**B**) views of the right middle ear. Complete obstruction of the mastoid cells is present without multiple bony sclerosis. A tympanic tube through the thickened eardrum has been placed. Obstruction of the epitympanum without bony erosion of the scutum is present.

**Figure 9:**
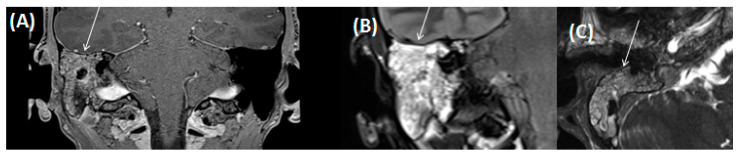
MRI of a 17-year-old male patient (same patient as shown in Figure 5 for CT) with coronal view for contrast-enhanced T1-weighted (**A**); coronal FLAIR (**B**); and axial CISS sequences (**C**). Note the complete obstruction of the mastoidal cells on the right side (arrow) as seen on the contrast-enhanced T1-weighted sequence (**A**) with discrete contrast enrichment of the mastoidal system. Total fluid obstruction is also demonstrated on FLAIR (**B**) and CISS sequences (**C**).

**Figure 10:**
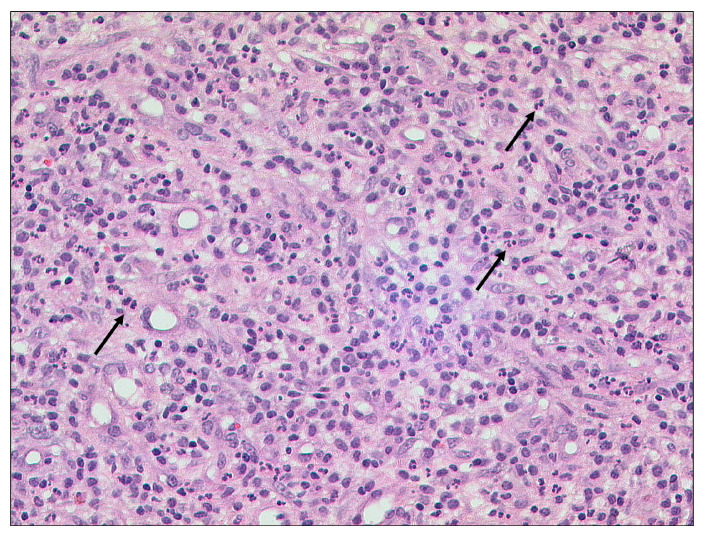
Acute and chronic mastoiditis, HE, original magnification 200:1. Granulation tissue with a mixed inflammatory infiltrate including numerous neutrophilic granulocytes (arrows).

Imaging with CT can show displacement of the mastoid cells with adjacent bony resorption (Figure 8). In contrast, accumulation of pus, especially in the mastoid cells, can occasionally be better delineated using MRI with hypointense signal on T1-weighted and hyperintense signal on T2-weighted imaging, usually demonstrating intermediate or strong contrast enhancement (Figure 9) [52,53]. Signs of adjacent meningeal affection are possible. The most important differential diagnoses to be considered are bone tumors and entities arising from lymphatic tissue. Histopathologically, acute mastoiditis is characterized by an infiltrate of neutrophilic granulocytes [54]. Most cases of mastoiditis show a variable combination of acute and chronic inflammation (Figure 10).

#### 3.2.3. Tympanosclerosis

**Figure 11:**
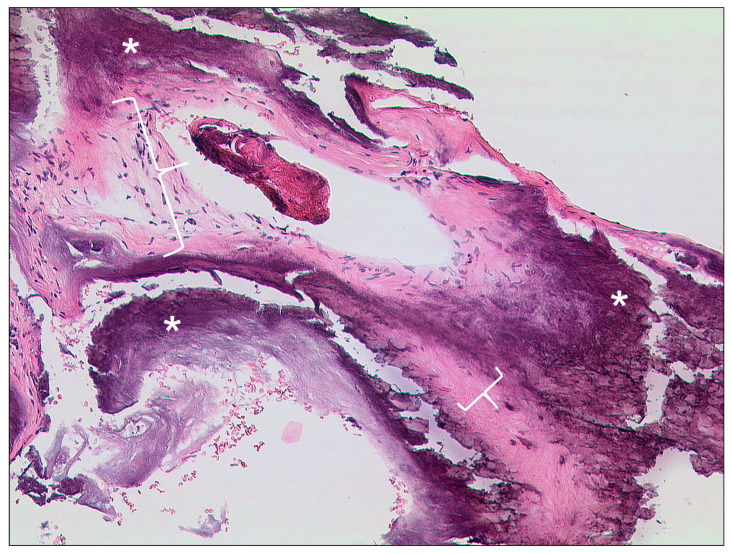
Tympanosclerosis, HE, original magnification 100:1. In this case of tympanosclerosis a dense fibrosis and hyalinazation (brackets) of connective tissue with massive calcification (asterisks) is seen.

**Figure 12:**
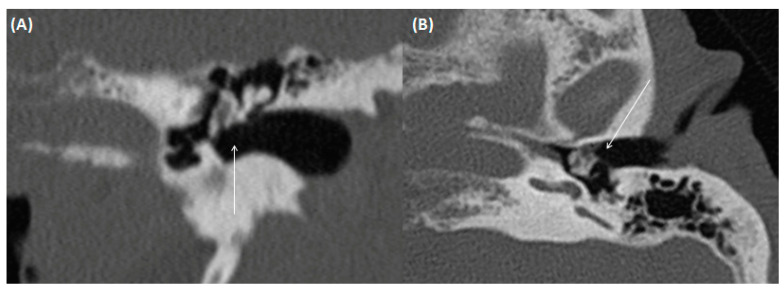
CT of a 73-year-old female patient with a history of chronic mastoiditis. Note the thickening of the eardrum and the adjacent ligaments of the ossicular chain as a sign of tympanosclerosis in coronar (**A**) and transversal (**B**) figure.

After repetitive middle ear infections, sclerosis and ossification of the ossicular chain and the supporting ligamentous structures of the middle ear can occur [55,56]. Clinically, scarring in the middle ear can be observed and a history of multiple middle ear infections can be indicative [57]. Diffuse hyaline formation and calcium deposits can be observed in specimen for histopathology (Figure 11) [57]. For imaging, CT is important and can show thin, tiny calcifications of the ossicular chain and the adjoining ligamenta mallei, the ligamentous incudis superius and posterius, as well as the ligamentum annulare stapedis (Figure 12) [56,58]. The extent can vary greatly from light calcifications to confluent bone-like plates. To avoid pitfalls, attention should be paid to calcifications, which can simulate parts of the ossicular chain.

A diagnostic benefit is not achieved by administration of contrast medium or MRI. Congenital malformations of the middle ear chain must be considered as a differential diagnosis. Therapeutically, evacuation with partial or complete replacement of the ossicular chain can be required in the form of tympanoplasty. In more extensive cases, repairment of the damaged eardrum is necessary in the form of myringoplasty, thus a precise preoperative imaging evaluation in correlation with hearing tests is necessary [59].

#### 3.2.4. Cholesteatoma of the Middle Ear

**Figure 13:**
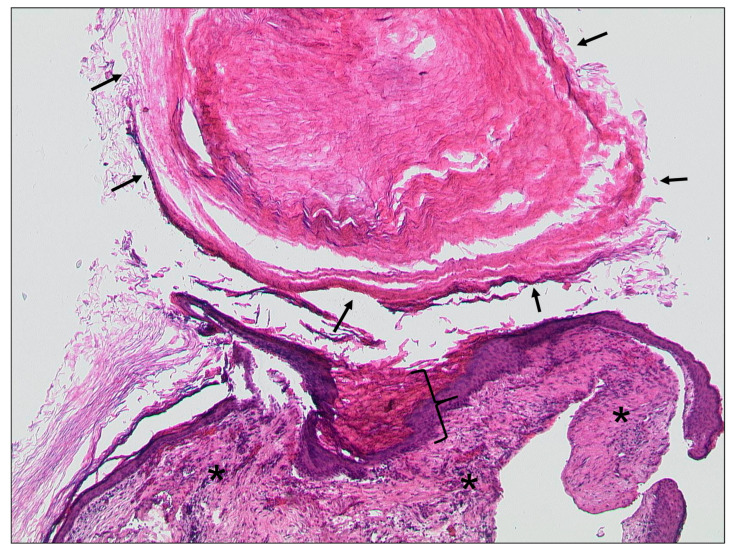
Cholesteatoma, HE, original magnification 50:1. Keratinizing squamous epithelium (bracket) with accumulated keratin (arrows) and associated chronic inflammation in the subepithelial connective tissue (asterisks).

Cholesteatoma as a chronic inflammatory disease can also affect the middle ear. The etiology has not yet been conclusively clarified [60]. Histopathologically, it is characterized by keratinizing squamous epithelium (Figure 13) [28,61]. A migration from the tympanic membrane into the different parts of the inner ear can be observed. The most common localization affected is the epitympanum with localization in the Prussak’s space [60].

In general, two types of acquired cholesteatoma can be differentiated: pars flaccida cholesteatoma and pars tensa cholesteatoma. An overview is given in Table 2.

**Figure 14:**
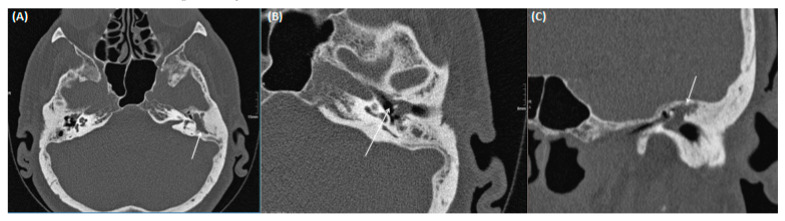
A 66-year-old male patient with pars flaccida cholesteatoma. Transversal (**A**,**B**) and coronal (**C**) CT using bone windowing. Complete soft tissue displacement of the epi-, meso-, and hypotympanum, but without bony destruction of the adjacent bony borders can be identified. Thickened tympanic membrane and beginning dissolution of scutum and ossicular chain are also present.

**Figure 15:**
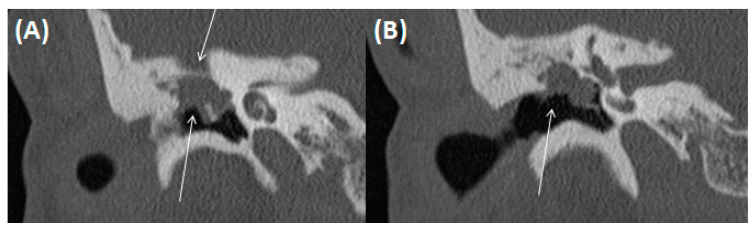
A 51-year-old male patient with cholesteatoma. Coronal CT using bone windowing. A hypodense mass is present in the epitympanum (arrows) with bony destruction of the scutum and beginning destruction of the ossicular chain. Note the beginning invasion towards the intracranial compartment. Figure (**A**,**B**) shows the middle ear in coronal view at different levels.

Typically, otoscopy shows a whitish tumor behind the eardrum, the continuity of which can also be interrupted. Furthermore, CT imaging is usually indicated preoperatively to diagnose the extent of the disease and rule out complications (Figure 14 and Figure 15). Here, a soft tissue proliferation with homogenous density of approximately 50 Hounsfield Units (HU) is registered, and bony erosion of the adjacent boundaries can be seen. There is no uptake of contrast media, but the surrounding granulation tissue may show hyperemia. Furthermore, MRI may delineate cholesteatoma as a T2-hyperintense, T1-hypointense lesion without contrast media uptake, but with prominent diffusion restriction [61,62]. Restricted diffusion on DWI is a leading imaging feature, which can help to identify the lesion, a potential recurrence, and differentiate it from other pathologies.

#### 3.2.5. Cholesterol Granuloma

**Figure 16:**
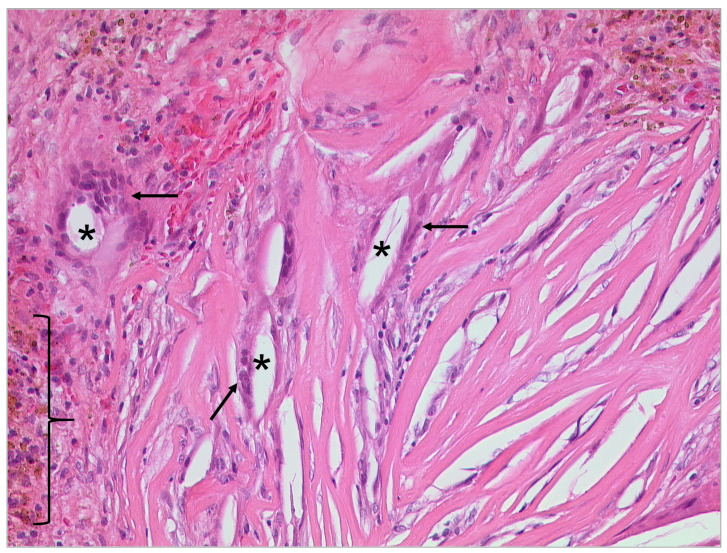
Cholesterol granuloma: Clefts from dissolved cholesterol crystals (asterisks), surrounded by multinuclear foreign body giant cells (arrows) in fibrous connective tissue. In the periphery, there are hemosiderin-laden macrophages (bracket). HE, original magnification 200:1.

In case of Eustachian tube dysfunction, there may be negative pressure or a vacuum phenomenon in the middle ear cavity, leading to mucosal edema and rupture of blood vessels with erythrocytosis [1,63]. The collapse of erythrocytes and tissue elements with cholesterol release can lead to a foreign body giant cell reaction [1], which is reflected in histopathology by a foreign body reaction, giant cell reaction to cholesterol crystals, and presence of hemosiderin derived from the rupture of the erythrocytes (Figure 16) [64]. Typically, it can be registered in the middle ear, mastoid, and petrous apex [64]. It is also referred to as cholesterol cyst, blue-domed cyst, chocolate cyst, or chocolate ear [1].

Mostly younger patients in their 20–30s are affected. Symptoms depend on localization. If it is located in the middle ear, patients typically present with a blue tympanic membrane, indicating hemotympanum with or without hearing loss. In case of localization in the petrous apex, cholesterol granulomas are mostly asymptomatic incidental findings; however, in rare cases, cranial nerve dysfunction can be registered [65].

**Figure 17:**
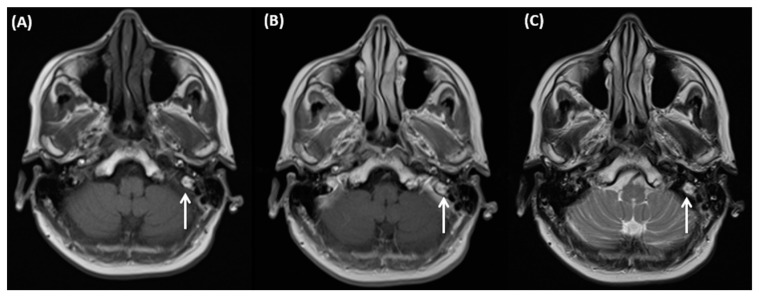
A 68-year-old female patient with incidental finding of a cholesterol granuloma located at the transition of the medial part to the apex of the left temporal bone (arrows). Axial T1-weighted MRI without (**A**) and with (**B**) administration of contrast agent. Axial T2-weighted imaging also shows the lesion as hyperintense (**C**).

On MRI, the characteristic finding is a hyperintense signal on unenhanced T1-weighted images due to the presence of blood products, which persist on fat-suppressed sequences (Figure 17). On T2-weighted images, signal intensity is usually heterogeneously hyperintense [1]. No contrast enhancement should be registered. The most important differential diagnosis is entrapment of fluid without superinfection. In case of symptomatic problems a surgical resection of the cyst is recommended. A potential surgical excision must include the cyst wall. Here, imaging could be helpful for operative planning; otherwise, differential diagnosis could be excluded.

### 3.3. Inner Ear

#### 3.3.1. Tympanogenic Labyrinthitis

There are many causes for labyrinthitis. Infections caused by bacteria or viruses are among the most common causes, but labyrinthitis can also arise from traumatic or postoperative changes [66]. It may occur when the natural defensive barriers of the middle ear are penetrated, and infection can involve the round window or oval window [66]. Typically, the infection or spread of toxins occurs from the middle ear to the inner ear via either the round window or oval window. In case of viral labyrinthitis that can present after a viral respiratory tract infection, the spread is hematogenous.

Widespread use of antibiotics in the management of otitis media has significantly reduced the incidence of labyrinthitis today [66]. Tympanogenic labyrinthitis can be secondary to middle ear disease, so it is typically unilateral. Sensory neural hearing loss or vertigo can be registered. An unremarkable otoscopic finding combined with progressive hearing loss are typical findings [66].

Imaging is performed to delineate the extent of the disease, particularly in the case of associated meningitis. However, it is important to notice that imaging in the initial phase can show false-negative results [15]. Contrast medium uptake in the vestibular system can be a leading finding on MRI. The signal on T1-weighted imaging is usually unremarkable, while signal intensity on T2-weighted images can be increased. Associated meningitis can be assessed using fluid-attenuated inversion recovery (FLAIR) or contrast-enhanced T1-weighted sequences. Furthermore, T2-weighted imaging has been shown to be sensitive regarding the identification of the fibrous stage, before the labyrinth is completely obstructed by bone [15]. In the acute and subacute phases, only few changes might be observable on CT images. Increased ossifications become apparent only after the disease has persisted for a long time. Intravestibular hemorrhages or tumors (e.g., schwannomas) must be considered for differential diagnosis [67]. A major differential diagnosis in cases of severe labyrinthitis ossificans is complete labyrinthine aplasia, which is characterized by the absence of the cochlear promontory [15].

#### 3.3.2. Otogene Purulent Meningitis

In addition to sinus vein thrombosis, purulent meningitis is one of the most common complications of inflammatory processes in the middle ear with cranial involvement. Particularly, in the absence of an obvious cause of bacterial meningitis, an otogenic focus must be considered. An MRI examination should be the preferred imaging approach [21,68].

## 4. Complications

There are mainly four mechanisms of extension of the infection in the middle ear and temporal bone: per continuitatem by preformed pathways, osseous erosions, thrombophlebitis, and hematogenous seeding [69]. When local regional inflammation cannot be contained, the suppuration under pressure causes local osteoclastic osseous resorption in all directions, and causes intratemporal or intracranial complications [69]. With the wide availability of antibiotics, the percentage of intracranial complications from otitis has decreased [69]. The most common early symptoms are fever and headache, sometimes combined with altered mental status and nuchal rigidity [69,70]. Furthermore, spread is possible over communicating veins and venules in the middle ear to the dura with vessels in the subarachnoid space and brain parenchyma. Microorganisms can spread through these veins and reach the subdural and subarachnoid spaces by mechanisms of thrombophlebitis and periphlebitis [69].

### 4.1. Gradenigo’s Syndrome

In the special form of Gradenigo’s syndrome there is a triad of otitis media, trigeminal neuralgia, and ipsilateral irritation of the sixth cranial nerve (abducens nerve palsy) [71,72]. It is named after the person who first described it, Guiseppe Gradenigo [73]. The diagnosis is often carried out with a delay, and with the increasing availability of antibiotics, the appearance of the disease is rare in industrialized nations. Even if acute otitis belongs to the classic triad, cases in connection with cholesteatomas and chronic otitis media have been described [73]. Therapy after the onset is often protracted with the administration of antibiotics over a longer period of time. Additional surgical repair of the mastoid is usually required. Imaging findings include bone destruction and opacifications of the mastoid air cells with or without extensive fluid in the left middle ear [74,75]. Changes in bone structures include erosive lysis with ill-defined irregular edges in the petrous apex [76]. The characteristic feature is permeative destruction of the bony structures in this region; however, bone loss occurs in later disease stages [76].

**Figure 18:**
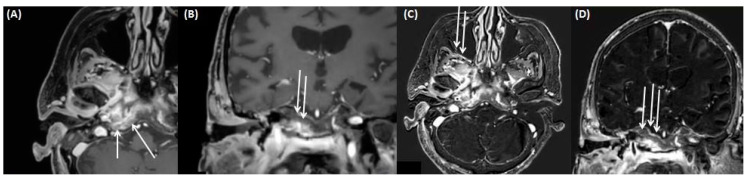
A 84-year-old female patient after mastoidectomy and signs of inflammation of the petrous apex and the skull base with involvement of the paths of the trigeminal and facial nerves, as shown on axial (**A**) and coronal (**B**) contrast-enhanced T1-weighted images, provided together with axial (**C**) and coronal (**D**) subtraction images (as derived from non-contrast-enhanced and contrast-enhanced T1-weighted images).

Signs of petrous apicitis involving the paths of the trigeminal, facial, vestibulocochlear, and abducens nerves have been registered [76]. Here, MRI is useful for the assessment of inflammatory soft tissue changes (Figure 18) [76].

### 4.2. Intracerebral Abscess/Emphysema/Meningitis

**Figure 19:**
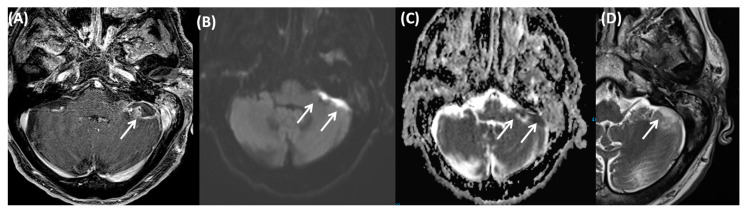
An 82-year-old male patient with intracranial infratentorial abscess after otomastoiditis. The abscess can be identified on T1-weighted fat-saturated sequences after contrast agent application (**A**), and can present with local signs of a meningeal irritation and demarked diffusion restriction (**B**,**C**). Axial T2-weighted imaging (**D**) shows intermediate intensity of the abscess.

Particularly in children, bacterial meningitis or subdural empyema can occur due to the more frequent occurrence of middle ear infections. Meningitis as a rare complication of mastoiditis results from hematogenous spread most of the time. In addition to otitis media with odontogenic cause, otherwise uncomplicated sinusitis can also be a trigger [77]. In addition to meningitis, an intracerebral abscess can be a serious complication of an inflammatory process in the middle ear [78,79]. Notably, this is usually located in close spatial proximity to the otogenic focus and most frequently in the temporal lobe or the cerebellum (Figure 19) [80]. Meningitis is defined as an inflammation of the subarachnoid space including arachnoidea and pia mater, and it can be caused by an infectious or noninfectious process. In meningitis, leptomeningeal enhancement can be registered on contrast-enhanced MRI. Arterial narrowing can potentially be registered with or without a hyperintense signal in sulci or cerebrospinal fluid (CSF)space on FLAIR sequences [78,79,80].

### 4.3. Cerebral Venous Sinus Thrombosis

Cerebral venous sinus thrombosis is a rare complication with possible otogenic thrombosis of the sigmoid and transverse sinus [81,82]. Dural venous thrombophlebitis is known to result from an extradural abscess in more than half of all cases [69]. A less common mechanism for dural thrombosis may be osteothrombophlebitis [69]. Pathophysiologically, this leads to inflammatory involvement of the dura and the adjacent outer wall layers of the sinus. The thrombosis can then spreads to the adjacent sinuses and extends caudally. It should be emphasized that it is an infectious thrombus with rapid onset of sepsis. Mortality is still around 5–10%, even in case of timely diagnosis [81]. Imaging with CT or MRI is obligatory, whereas MRI is preferred adequately detect simultaneous intracerebral complications [83]. Classically, contrast medium surrounds the thrombus with negative contrast, and simultaneously there is a strong contrast medium uptake in the sinus wall as an indication of the underlying infection. In addition to antibiotics, mastoidectomy and intraoperative evacuation of the sinus can be necessary therapeutical interventions. Permanent anticoagulation is mandatory. Another aggressive therapeutic option is caudal vein ligation to prevent the spread of infection [80].

### 4.4. Otitic Hydrocephalus

Hydrocephalus is a rare intracranial complication of otitis media [84,85]. It is characterized by increased CSF pressure with normal CSF composition and no focal neurological deficits. It was first described by Symonds [86]. In most cases, children and adolescents are affected [87]. The exact pathophysiology of this rare disease has not been finally clarified. An increased production or reduced absorption of CSF following obstruction of the lateral sinus, which affects cerebral venous outflow or extension of thrombus material to the superior sagittal sinus impeding CSF resorption, are discussed [87]. Signs of increased intracranial pressure can be registered, including prominent subarachnoid spaces surrounding the optic nerves and the finding of an “empty sella”. Diagnosis is based on the course of imaging using CT or MRI [86,87]. Furthermore, imaging could help to exclude associated brain abscess [87]. Symptoms are unspecific such as headache, nausea and vomiting. A delayed onset long time after chronic middle ear disease is possible.

### 4.5. Ramsay Hunt Syndrome

Ramsay Hunt syndrome (RHS) is an infectious disease caused by the Herpes Zoster virus. It is also called Herpes Zoster Oticus. Ear involvement in Herpes Zoster viral infection is registered in 12% of cases of peripheral facial palsy [88,89]. Prognosis for facial palsy is poorer in RHS than in idiopathic forms; only in 10% of complete facial palsy the symptoms are resolved completely [90].

The syndrome was first described by J. Ramsay Hunt in 1907 [88,90]. The main clinical symptoms are facial palsy, inner ear dysfunction, and, as a leading symptom, periauricular pain. In particular, cutaneous findings of Herpes Zoster Oticus in the innervation area of the trigeminal nerve including sensitivity disturbances point to the diagnosis of RHS [91]. Following peripheral facial nerve paresis, taste disturbances and reduction in tear secretion are the most common findings. All other findings are less common and less specific, especially lesion of the acoustic nerve including hearing loss and cases of vertigo [91]. Typically, MRI shows an increased enhancement of the facial nerve. Furthermore, MRI can help to differentiate between Bell’s palsy of the peripheral facial nerve and RHS. Early diagnosis is crucial to support rapid initiation of a combination therapy with systemic steroids and antiviral agents [92].

## 5. Conclusions

Inflammatory pathologies of the temporal bone and middle ear are common, and imaging is indicated to assess the extent of disease and complications. The causal entities for disease are diverse, and a systematic approach for classification using anatomical landmarks and clinical features is necessary. For a radiologist, it is always advisable to look at the images systematically, e.g. from the outside inwards. All structures must always be considered and evaluated. In general, the presence of CT and MRI facilitates the synopsis of findings, and oftentimes more than one imaging modality is required.

Overall, this review provided a comprehensive point-to-point comparison of the radiological and histological features of the wide spectrum of inflammatory diseases of the temporal bone and middle ear. We first introduced the anatomical and imaging fundamentals in context with a review of the current literature, and highlighted the clinical implications of imaging modalities. A punctual outlook regarding current trends and future directions was provided as well.

## Figures and Tables

**Table 1:** Overview of the most common differential diagnoses for inflammatory diseases of the petrous bone and middle ear and their mimics.

External auditory canal	Otitis externa maligna [11] Cholesteatoma of the external ear canal [11] Auditory canal exostosis [12] Keratosis obturans [13]
Middle ear and mastoid	Acute otitis media [14] Chronic otitis media [14] Mastoiditis [15] Tympanosclerosis [15] Cholesteatoma of the middle ear [16] Cholesterol granuloma [15] Inflammatory pseudotumor [17] Wegner granulomatosis [18] Petrous apicitis [19]
Inner ear	Tympanogenic labyrinthitis [20] Otogene purulent meningitis [21]

**Table 2:** Overview of types of cholesteatoma.

	Localization	Ossicular Displacement	Extension to Mastoid Antrum
Pars flaccida cholesteatoma	Prussak’s space	Medial	Lateral to incus
Pars tensa cholesteatoma	Posterosuperior retraction	Lateral	Medial to incus

## Abbreviations

CSF	cerebrospinal fluid space
CT	computed tomography
DWI	diffusion weighted imaging
EAC	external auditory canal
EACC	external ear canal cholesteatoma
EPI	echo planar imaging
FLAIR	Fluid-attenuated inversion recovery
HE	heamatoxylin-eosin
HU	Hounsfield units
MRI	Magnetic resonance imaging
RHS	Ramsay Hunt syndrome
